# Comparative Radiologic and Morphologic Analysis of Posterolateral Fusion and Percutaneous Pedicle Screw Fixation for Thoracolumbar Junction Burst Fractures

**DOI:** 10.3390/jcm14186379

**Published:** 2025-09-10

**Authors:** Hyung-Rae Lee, Minseung Kang, Jae Min Park, Jae-Hyuk Yang

**Affiliations:** 1 Department of Orthopedic Surgery, Korea University Anam Hospital, Seoul 02841, Republic of Korea; drhrleeos@gmail.com; 2 College of Medicine, Korea University, Seoul 02841, Republic of Korea; vickyk03@korea.ac.kr (M.K.); kikara02@korea.ac.kr (J.M.P.)

**Keywords:** thoracolumbar burst fracture, percutaneous fixation, posterolateral fusion, morphologic grade, sagittal alignment, vertebral body restoration, minimally invasive spine surgery

## Abstract

**Background/Objectives:** Thoracolumbar burst fractures often require surgical stabilization. Although posterolateral fusion (PLF) has been traditionally used, percutaneous posterior fixation (PPF) without fusion has emerged as a less invasive alternative. However, comparative data specifically addressing PPF and PLF are limited. This study aimed to compare the radiological and perioperative outcomes of PPF and PLF for thoracolumbar burst fractures. **Methods:** This retrospective cohort study analyzed 61 patients with T11–L2 burst fractures (PPF, 28; PLF, 33). Radiological parameters included local and global sagittal alignment and vertebral height ratio. Fracture morphology was assessed using a structured grading system based on anterior height ratios. Perioperative variables were also assessed. Statistical significance was set at *p* < 0.05. **Results:** PPF demonstrated significant advantages in operative time (160.7 min vs. 205.8 min, *p* < 0.01) and blood loss (165 cc vs. 317 cc, *p* < 0.01), with a shorter hospitalization time. PPF achieved outcomes comparable to PLF in global alignment and anterior height restoration. The PLF group showed greater local kyphotic angle correction (−7.77° vs. −1.53°, *p* = 0.01), whereas the PPF group showed significantly higher postoperative posterior height ratio (*p* = 0.02). Changes in morphological grades, assessed using the anterior height ratio-based grading system, showed similar patterns of improvement in both groups. All implant removals were performed due to patient-reported discomfort. **Conclusions:** PPF yielded radiological outcomes comparable to PLF in the treatment of thoracolumbar burst fractures. The use of a morphological grading system provided a structured descriptive tool to evaluate surgical impact, though its utility remains exploratory and requires further validation.

## 1. Introduction

Thoracolumbar junction burst fractures represent a biomechanically challenging subset of spinal injuries that occur at the transition zone between the relatively rigid thoracic spine and the more mobile lumbar spine [1]. The optimal surgical strategy remains an area of investigation, particularly regarding the choice between open and minimally invasive posterior fixation techniques and the necessity of fusion [2].

Traditionally, posterolateral fusion (PLF) has been employed to restore spinal stability, correct deformity, and promote bony union [3,4,5,6]. The addition of fusion is believed to enhance long-term outcomes by encouraging solid arthrodesis, thereby reducing the risk of implant failure or kyphotic collapse [7]. In contrast, percutaneous posterior screw fixation (PPF) has gained popularity as a minimally invasive alternative, offering reduced surgical morbidity by minimizing soft tissue disruption [8]. However, PPF is typically performed without formal bony fusion, relying instead on hardware stabilization during the fracture healing period [3].

Despite the growing literature comparing open versus percutaneous screw placement, most existing studies have not clearly distinguished between fusion and non-fusion techniques within these approaches [9]. More importantly, there is a conspicuous lack of studies that directly compare PPF (non-fusion) with PLF (open + fusion) in the specific context of traumatic thoracolumbar junction burst fractures [10]. This distinction is clinically significant, as fusion and non-fusion constructs differ not only in surgical invasiveness but also in their biological healing goals and mechanical stability over time.

While numerous prior investigations have addressed screw placement techniques or fusion outcomes in degenerative spine conditions, few, if any, have directly addressed the specific clinical question of whether PPF can achieve comparable outcomes to PLF in traumatic burst fractures [7]. Given the increased morbidity associated with open fusion procedures and the evolving preference for less invasive solutions, this gap in the literature underscores the need for targeted comparative studies [3].

Therefore, the present study aims to fill this critical void by conducting a comprehensive, controlled comparison of PPF (non-fusion) and PLF (open + fusion) in patients with thoracolumbar junction burst fractures [3]. The investigation includes detailed evaluations of sagittal alignment (global and local), vertebral morphology, and perioperative outcomes, offering a nuanced understanding of the trade-offs between invasiveness, radiological correction, and long-term clinical stability. This approach not only addresses a previously unexamined question but also holds the potential to inform future clinical decision-making and refine treatment guidelines for this complex injury [2].

## 2. Materials and Methods

### 2.1. Patient Selection

This retrospective cohort study was approved by the Institutional Review Board (IRB) of Korea University Anam Hospital (IRB number: 2025AN0085) and was conducted in accordance with the principles of the Declaration of Helsinki and the STROBE (Strengthening the Reporting of Observational Studies in Epidemiology) guidelines.

Patients who underwent posterior instrumentation for thoracolumbar junction (T11–L2) burst fractures between January 2015 and August 2022 were screened for eligibility, ensuring a minimum follow-up duration of 24 months for all included cases. Surgical candidacy was determined according to the Thoracolumbar Injury Classification and Severity Score (TLICS), and only patients with scores ≥ 4 were considered for operative treatment. Posterior column disruption was defined as both posterior ligamentous complex (PLC) injury and spinous process fracture, as confirmed on preoperative magnetic resonance imaging (MRI) or computed tomography (CT) [11].

In this study, spinous process fracture was interpreted as a potential indirect marker of ligamentous compromise or failure of the posterior tension band. This approach aligns with prior research emphasizing that PLC integrity, encompassing the supraspinous ligament, interspinous ligament, ligamentum flavum, and facet capsule, is a critical determinant of spinal stability and surgical decision-making in thoracolumbar burst fractures [12,13]. Accordingly, comprehensive preoperative MRI evaluation of PLC integrity was routinely performed to guide the treatment strategy, consistent with the recommendations of the TLICS system [2,14].

**Figure 1:**
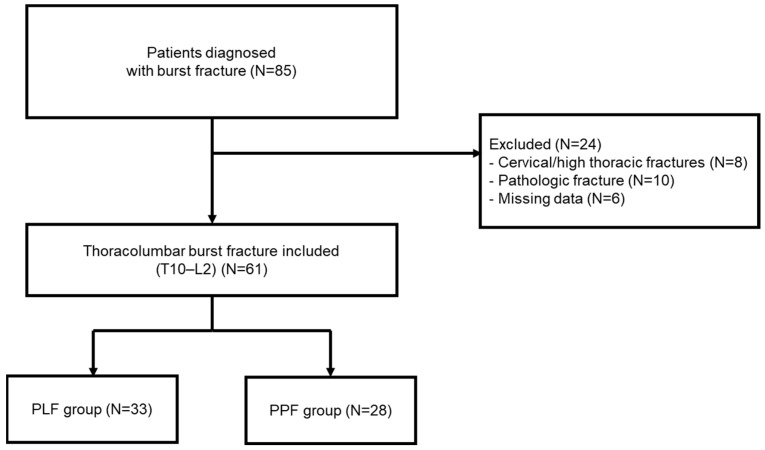
Flow chart.

A total of 85 patients with thoracolumbar fractures were identified. After applying exclusion criteria—including cervical or high thoracic fractures (*n* = 8), pathologic or osteoporotic fractures (*n* = 10), and incomplete radiologic or clinical data (*n* = 6)—a final total of 61 patients with T11–L2 burst fractures were included in the study (Figure 1).

The patients were divided into two groups according to the surgical approach: the PLF group (posterolateral fusion using the open technique, *n* = 33) and the PPF group (percutaneous posterior screw fixation using the minimally invasive technique, *n* = 28). All procedures were performed by two senior spine surgeons. The choice between PLF and PPF was not based on a standardized algorithm but rather reflected temporal practice patterns and surgeon preference. Earlier in the study period, PLF was more frequently performed as the conventional open technique, whereas PPF was increasingly adopted in later years following the introduction of minimally invasive instrumentation.

### 2.2. Surgical Procedure

PLF and PPF were performed using standardized techniques appropriate for each approach.

In the PLF group, a midline posterior incision was made, followed by bilateral dissection and retraction of the paraspinal muscles to expose the posterior spinal elements. Pedicle screws were inserted under direct visualization. In all cases, decortication of the transverse processes was performed, and local bone grafts were placed in the intertransverse space to achieve posterolateral fusion. Laminectomy was performed as needed, depending on the presence of neurological compression.

In the PPF group, multiple small skin incisions were made laterally at the pedicle entry sites under fluoroscopic guidance. Guide wires (K-wires) were inserted percutaneously into the pedicles, followed by sequential muscle dilatation to create working channels. Cannulated pedicle screws were inserted over the guidewires using a muscle-splitting approach. The rods were passed subfascially and connected percutaneously with fluoroscopic assistance. Formal fusion or bone grafting was not performed in the PPF group.

All procedures were performed by experienced spine surgeons, using consistent protocols within each surgical category.

### 2.3. Radiological Measurement

Radiological assessments were conducted to evaluate sagittal spinal alignment and vertebral morphology before and after surgery. All measurements were performed by two independent spine surgeons using standardized digital radiographic software, and the average values were used for analysis.

Sagittal alignment was categorized into two domains—local alignment and global alignment.

**Figure 2:**
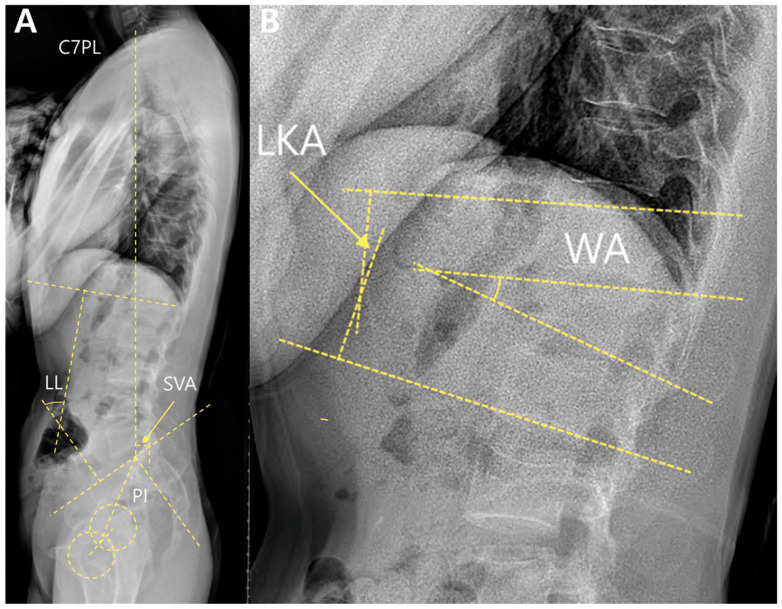
Measurements of spinal parameters. (**A**) Standing whole-spine lateral radiograph of a patient with an L1 burst fracture; (**B**) Magnified lateral view at the fracture level. The wedge angle (WA) was measured between the upper and lower endplates of the fractured vertebra, and the local kyphotic angle (LKA) was measured between the superior endplate of the vertebra above and the inferior endplate of the vertebra below. **Abbreviations:** C7PL, C7 plumb line; SVA, sagittal vertical axis; LL, lumbar lordosis; PI, pelvic incidence; WA, wedge angle; LKA, local kyphotic angle.

Local alignment included the local kyphotic angle (LKA) and wedge angles (WA). These were measured using standing whole-spine lateral radiographs (Figure 2).

The LKA was defined as the angle between the superior endplate of the vertebra above and the inferior endplate of the vertebra below the fractured segment. The WA was measured between the upper and lower endplates of the fractured vertebrae.

Global alignment parameters included the sagittal vertical axis (SVA), lumbar lordosis (LL), pelvic incidence (PI), and PI–LL mismatch. The SVA was defined as the horizontal distance between the C7 plumb line and the posterosuperior corner of the S1 endplate. LL was measured as the angle between the superior endplates of L1 and S1. PI is defined as the angle between the line perpendicular to the sacral endplate and the line connecting the midpoint of the sacral endplate to the femoral head axis. The PI–LL mismatch was calculated by subtracting LL from PI.

**Figure 3:**
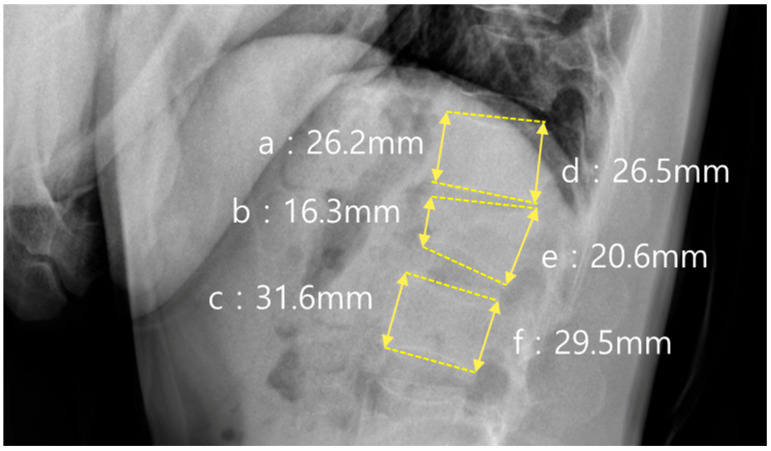
Measurements of vertebral body height ratios. Anterior and posterior vertebral body height ratios were calculated based on the fracture level using the formulas: Anterior ratio = (b × 2)/(a + c) × 100%, Posterior ratio = (e × 2)/(d + f) × 100%. In this figure, an L1 fracture is shown. The anterior body height ratio was 56.3%, and the posterior body height ratio was 73.8%: (**a**) a level above fracture (anterior); (**b**) fracture level (anterior); (**c**) a level below fracture (anterior); (**d**) a level above fracture (posterior); (**e**) fracture level (posterior); (**f**) a level below fracture (posterior).

In addition to sagittal alignment, the vertebral body morphology was evaluated using the anterior and posterior vertebral body height ratios, as shown in Figure 3. The anterior height ratio was calculated as (b × 2)/(a + c) × 100, where a and c represent the anterior vertebral body heights of the vertebrae above and below the fracture, respectively, and b represents the anterior height at the fracture level. The posterior height ratio was calculated using the formula (e × 2)/(d + f) × 100, where d and f represent the posterior vertebral heights of the adjacent vertebrae, and e represents the posterior height of the fractured vertebra.

All radiographic measurements were obtained using magnified digital images of standardized sagittal planes with correction for rotation and projection variation. In addition, MRI was routinely performed in all patients with thoracolumbar fractures to evaluate posterior ligamentous complex (PLC) integrity. PLC injury was defined as discontinuity or abnormal high signal intensity of the supraspinous/interspinous ligaments, ligamentum flavum, or facet joint capsules. Post-operative radiographs were obtained in the immediate postoperative period (at discharge or within days of surgery), at three months, at six months, and annually, when available. The final follow-up was defined as the 24-month postoperative visit, and radiological parameters at this time point were used for comparative analyses.

### 2.4. Morphologic Grading System

**Figure 4:**
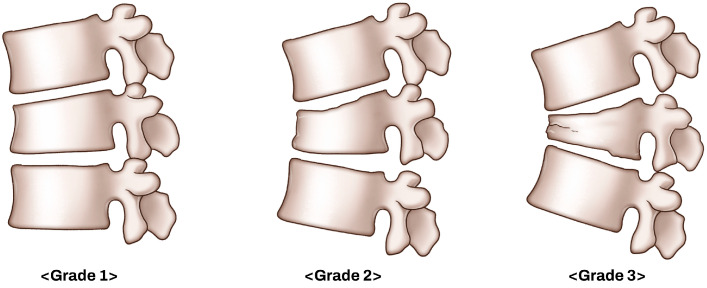
Grading system based on anterior vertebral body height ratio. Fracture severity was graded according to the anterior body height ratio at the fracture level. Grade 1 was defined as a ratio ≥ 80%, Grade 2 as a ratio between 60% and 80%, and Grade 3 as a ratio < 60%.

Fracture severity was further evaluated using a morphological grading system based on the anterior vertebral body height ratio at the fracture level. As illustrated in Figure 4, this system categorizes fractures into three grades according to the extent of anterior vertebral body collapse.

Grade 1 was defined as an anterior body height ratio of 80% or greater, indicating minimal compression. Grade 2 corresponded to a ratio of 60% to 80%, reflecting moderate compression. Grade 3 was defined as a ratio of <60%, representing severe anterior column collapse.

The selection of 60% and 80% as critical thresholds in our morphological grading system was informed by the established biomechanical and clinical criteria for vertebral stability and surgical outcomes. A vertebral body height ratio <60% (equivalent to >40% anterior height loss) has been widely recognized as a marker of instability in thoracolumbar fractures [15,16]. This aligns with surgical guidelines indicating that anterior height loss exceeding 40% disrupts load-bearing capacity, increasing the risk of progressive kyphosis and neurological compromise [17]. Notably, this >40% threshold is not part of the formal TLICS system; in our study, operative indication was determined by TLICS (≥4), while the anterior height-based grading was applied as an additional morphological severity descriptor.

Conversely, postoperative restoration of anterior vertebral height to ≥80% is associated with favorable outcomes [18,19]. Clinical studies demonstrate that achieving ≥80% height restoration correlates with improved pain relief, functional recovery, and reduced adjacent-level fracture risk [17]. Kyphoplasty data further support this threshold, showing that height restoration ≥80% restores spinal biomechanics to near-normal levels, minimizing sagittal imbalance and secondary complications. These evidence-based thresholds provide a reproducible framework for assessing the fracture severity and therapeutic success.

This grading framework allows for the intuitive classification of fracture severity using quantitative and radiographically reproducible parameters. The same anterior vertebral height measurements used in the morphological analysis were applied to the grading framework.

### 2.5. Statistical Analysis

All statistical analyses were performed using IBM SPSS Statistics (version 26.0; IBM Corp., Armonk, NY, USA). Continuous variables were expressed as mean ± standard deviation (SD) and compared between groups using independent *t*-tests or the Mann-Whitney U test for non-parametric data. Categorical variables are presented as frequencies and percentages and were analyzed using chi-square tests or Fisher’s exact tests, as appropriate. Statistical significance was set at *p* < 0.05 for all comparisons. This statistical framework ensured an objective and rigorous evaluation of intergroup differences in radiological and perioperative parameters.

## 3. Results

### 3.1. Demographic and Clinical Characteristics

A total of 61 patients with thoracolumbar junction burst fractures were enrolled in this study, including 33 who underwent open PLF and 28 who underwent PPF. Baseline demographic and clinical characteristics were comparable between the two groups, with no statistically significant differences observed (Table 1).

The mean age was 66.64 ± 11.99 years in the PLF group and 65.71 ± 16.98 years in the PPF group (*p* = 0.81). Sex distribution (PLF, 11:22; PPF, 11:17), BMI, and BMD T-scores did not differ significantly between groups. The prevalence of chronic kidney disease showed a nonsignificant trend toward a higher incidence in the PLF group (12.1% vs. 0%, *p* = 0.06). The use of osteoporosis medications before and after the fracture was not significantly different between groups.

### 3.2. Radiologic Characteristics: Fracture Level and Fixation Pattern

The radiological characteristics and instrumentation details are listed in Table 2. The distribution of the fracture levels was similar in both groups, with L1 being the most commonly affected vertebra (PLF, 42.4%; PPF, 42.9%). However, significant differences were observed in the fixation patterns. The PLF group showed a tendency toward longer constructs, with a greater proportion of cases involving two or more levels above (63.6%) and below (54.5%) the fractured segment. In contrast, the PPF group used single-level fixation (71.4%) and fixation below the fracture sites (75.0%) more frequently. These differences were statistically significant (*p* = 0.03 and *p* = 0.04, respectively). Importantly, fracture-level fixation was performed in all PPF cases (100.0%), whereas it was omitted in approximately 27% of PLF cases (*p* < 0.01). Although posterior column disruptions, including PLC injury and spinous process fracture, were more frequently observed in the PPF group (46.4% vs. 30.3%, respectively), the difference was not statistically significant (*p* = 0.19).

### 3.3. Perioperative Outcomes

Table 3 summarizes the perioperative outcomes and complications. Operative time was significantly shorter in the PPF group (160.71 ± 52.65 min) than in the PLF group (205.79 ± 66.31 min, *p* < 0.01). Likewise, estimated blood loss was markedly reduced in the PPF group (165.00 ± 13.74 cc) compared with the PLF group (317.27 ± 189.48 cc, *p* < 0.01). Length of hospital stay showed a borderline significant trend favoring PPF (11.00 ± 6.99 vs. 16.27 ± 13.03 days, *p* = 0.05). No statistically significant differences were found between the groups in terms of complication rates, including screw loosening (PLF, 24.2%; PPF, 17.9%), revision surgery, implant prominence, or implant removal.

### 3.4. Sagittal Alignment

The radiographic parameters related to sagittal alignment are summarized in Table 4.

Both the PLF and PPF groups showed improvements in LKA and WA after surgery. While no statistically significant differences were observed in the absolute preoperative and postoperative LKA values (*p* = 0.39 and *p* = 0.92, respectively), the amount of kyphotic angle correction (Post-Pre) was significantly greater in the PLF group (−7.77 ± 9.96°) compared to the PPF group (−1.53 ± 7.60°, *p* = 0.01). Changes in WA were not statistically different between the groups (*p* = 0.64).

Global sagittal parameters, including PI, LL, PI–LL mismatch, and SVA, did not show significant differences between the two groups in either preoperative or postoperative values or the amount of change over time (all *p* > 0.05).

### 3.5. Vertebral Morphology

The changes in vertebral body height ratios are presented in Table 5.

The anterior vertebral body height ratio increased postoperatively in both groups. There were no significant differences in preoperative (*p* = 0.28), postoperative (*p* = 0.65), or delta values (*p* = 0.58) between the PLF and PPF groups.

Posterior body height ratio was significantly higher in the PPF group postoperatively (90.24 ± 9.00%) compared to the PLF group (79.70 ± 21.20%, *p* = 0.02). However, the preoperative values (*p* = 0.27) and amount of change over time (*p* = 0.50) were not significantly different.

### 3.6. Morphologic Grading Outcomes

**Figure 5:**
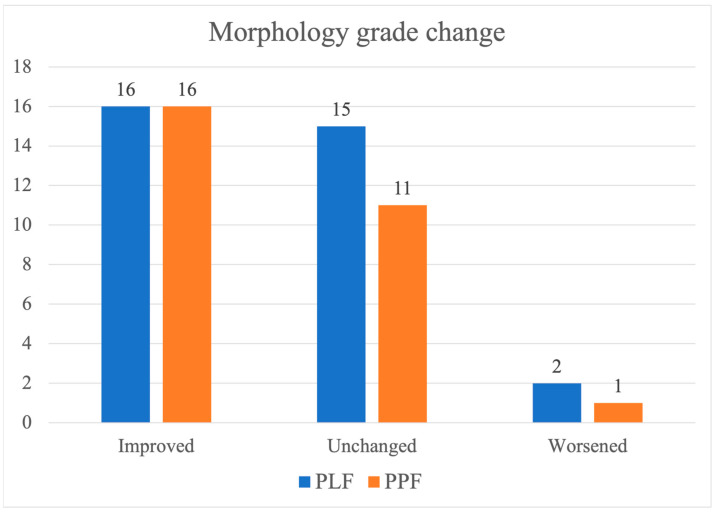
Changes in vertebral morphology grade after surgery. Bar chart showing postoperative changes in morphology grade in the PLF and PPF groups. In both groups, 16 patients showed improvement. The number of patients with unchanged morphology was 15 in the PLF group and 11 in the PPF group. Worsening of morphology was observed in 2 PLF patients and 1 PPF patient. Abbreviations: PLF, posterolateral fusion; PPF, percutaneous posterior fixation.

Vertebral fracture severity assessed using the morphological grading system based on the anterior body height ratio is summarized in Table 6 and Figure 5. Before surgery, the distribution of grades differed between the two groups, without statistical significance (*p* = 0.17).

In the PLF group, five patients (15.2%) were classified as grade I, 11 (33.3%) as grade II, and 17 (51.5%) as grade III. In the PPF group, three patients (10.7%) had grade I, 16 patients (57.1%) had grade II, and nine patients (32.1%) had grade III.

Following surgery, a shift toward lower grades was observed in both groups. In the PLF group, grade I increased to 15 patients (45.5%), grade II increased to 10 patients (30.3%), and grade III decreased to eight patients (24.2%). In the PPF group, grade I was observed in 13 patients (46.4%), grade II in 13 (46.4%), and grade III in two (7.1%). No statistically significant difference in the postoperative grade distribution was found between the two groups (*p* = 0.15).

As shown in Figure 5, 16 patients in each group showed improvement in fracture grade after surgery. The morphological grade remained unchanged in 15 patients in the PLF group and 11 patients in the PPF group. Grade worsening was observed in two patients (6.1%) in the PLF group and one patient (3.6%) in the PPF group. Subgroup analyses by an anterior height-based morphological grade (I ≥ 80%, II 60–80%, III < 60%) demonstrated consistent postoperative improvement across grades, with no significant between-group differences within grades.

**Figure 6:**
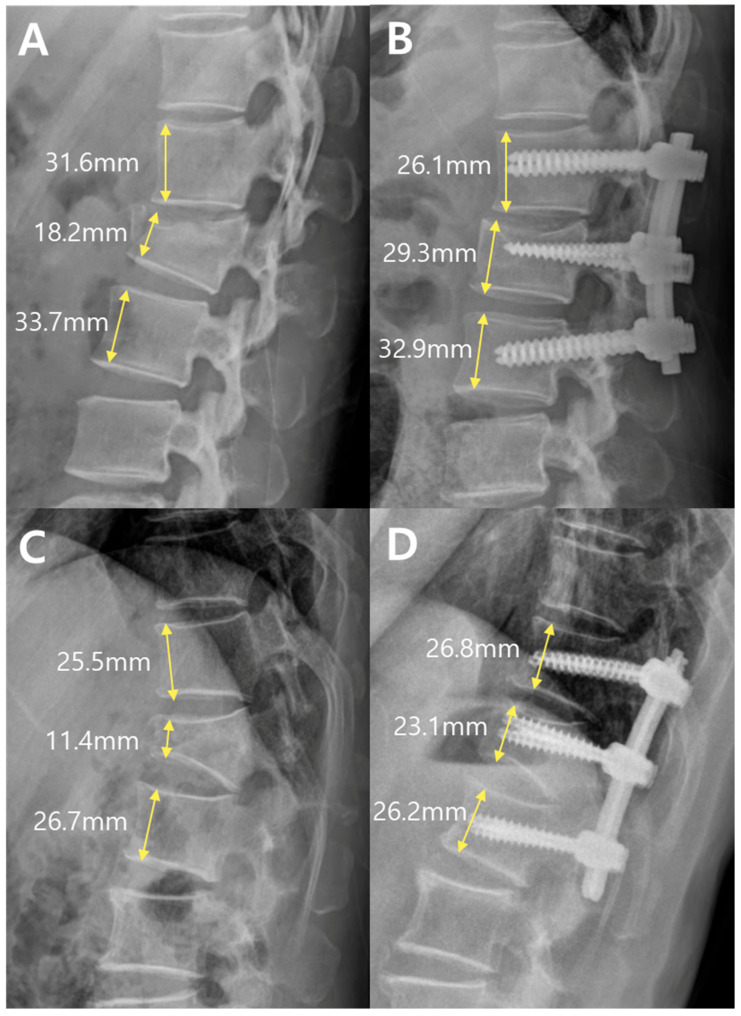
Representative patients from the PLF group (**A**,**B**) and the PPF group (**C**,**D**). (**A**,**B**) Preoperative (Pre) lateral radiograph from the PLF group showing an anterior body height ratio of 56%, and a postoperative (Post) radiograph showing an increased ratio of 99%. (**C**,**D**) Preoperative lateral radiograph from the PPF group showing an anterior body height ratio of 43%, and a postoperative radiograph showing an increased ratio of 87%.

Representative preoperative and postoperative radiographs of both groups are shown in Figure 6. These images visually demonstrate the anterior vertebral height restoration and screw construct placement. Notably, a substantial height recovery was observed even in the absence of bone grafting in the PPF case (D vs. C), highlighting the capacity of percutaneous fixation alone to support vertebral remodeling.

## 4. Discussion

This study demonstrated that PPF achieves outcomes comparable to traditional PLF in the treatment of thoracolumbar burst fractures while improving surgical efficiency [2,20,21]. Our hypothesis that PPF would provide equivalent radiological and morphological results despite omitting formal fusion was supported by multiple objective metrics, validating the clinical viability of fusion-free stabilization in appropriately selected cases [9,22,23].

While earlier studies compared either the surgical approach (open vs. percutaneous) or fusion status (fusion vs. non-fusion) in isolation [20,21], our study uniquely integrated both variables. Previous analyses have often conflated these factors, as open procedures typically involve fusion, whereas percutaneous techniques generally do not [24,25].

PPF demonstrated significant perioperative advantages, including shorter operative time (160.71 vs. 205.79 min, *p* < 0.01), reduced blood loss (165.00 vs. 317.27 cc, *p* < 0.01), and shorter hospitalization (11.00 vs. 16.27 days, *p* = 0.05; Table 3). These differences are largely attributable to its minimally invasive nature and avoidance of bone grafting, contributing to overall reductions in surgical burden [26,27]. Shorter operative duration is associated with a lower incidence of intraoperative and postoperative adverse events such as surgical site infection, wound dehiscence, and venous thromboembolism, with each 15-min increase in operative time correlating with a proportional rise in complication risk [28]. Moreover, reduced blood loss decreases transfusion requirements and the attendant risks of transfusion-related complications, including immunomodulation and increased infection rates, which have been linked to worsened perioperative morbidity and prolonged recovery in spine surgery patients [29,30]. Therefore, choosing PPF may represent an optimal option for minimizing perioperative risks and enhancing early postoperative recovery in thoracolumbar junction fracture patients.

In terms of spinal alignment, both groups maintained comparable global sagittal profiles postoperatively (PI, LL, PI–LL mismatch, and SVA; Table 4). Although PLF achieved significantly greater LKA correction (ΔLKA: −7.77° vs. −1.53°, *p* = 0.01), this outcome reflects the mechanical advantages inherent to open instrumentation [31,32]. The use of rod contouring and intraoperative compression–distraction maneuvers in PLF allows surgeons to apply direct corrective forces, which can effectively restore anterior height. However, this technique often results in a relative reduction in the posterior height. Consistent with this mechanism, our results showed no significant difference in the posterior vertebral body height ratio between the groups preoperatively, but a significantly lower value was observed in the PLF group postoperatively (79.70% vs. 90.24%, *p* = 0.02; Table 5) [31,33]. This pattern is expected to represent a natural biomechanical trade-off during kyphosis correction.

Several observations may explain the relative differences in local alignment outcomes between the two groups. Unlike PLF, PPF cannot manipulate rod contouring freely, as correction is largely constrained by the fixed screw positions [31,34]. Nonetheless, in the context of burst fractures, intraoperative prone positioning often facilitates passive vertebral height restoration through natural recoil, especially in non-fused segments [35]. Our findings indicate that while the local angular correction in PPF was less pronounced, the overall radiologic and morphologic outcomes were comparable to those of PLF, suggesting that this degree of correction may still be adequate in appropriate clinical contexts, particularly those without severe angular deformities [36,37].

In this context, intraoperative positioning may serve as a compensatory mechanism for limited angular control in PPF. Prone placement with chest and pelvic bolstering has the potential to induce ligamentotactic forces, allowing partial anterior height restoration even prior to instrumentation. Through careful adjustment of the table posture and bolster configuration, incremental kyphotic correction may be achievable [38,39]. This effect could help account for the comparable alignment outcomes observed between the groups and aligns with previous studies suggesting a biomechanical role for prone positioning in burst fracture management [40].

Despite these differences in local deformity correction, global sagittal alignment remained comparable between the two groups. Maintaining sagittal alignment is critical in spinal surgery, as malalignment has been associated with chronic back pain, fatigue, and functional disability [41,42]. In our analysis, both PLF and PPF successfully preserved global sagittal balance postoperatively with comparable SVA and PI–LL mismatch values (Table 4). While PLF achieved greater local angular correction, this may hold limited clinical relevance in patients with relatively preserved preoperative alignment [43]. Conversely, PPF’s ability to preserve posterior vertebral height and avoid overcorrection may contribute to sustained segmental and global balance over time [44]. These findings support the notion that percutaneous instrumentation, when appropriately applied at the fracture level, can provide adequate control over sagittal alignment without the need for formal fusion or extended constructs [45].

Moreover, previous studies have emphasized that global sagittal balance is a key predictor of long-term functional recovery and pain outcomes in patients with thoracolumbar junction fractures [46,47,48]. Given this context, the similar global alignment profiles observed in our cohort may imply equivalent clinical trajectories between PLF and PPF, although further studies are warranted because of the absence of direct clinical outcome evaluation.

To further enhance the objective evaluation of vertebral height restoration, we introduced a novel anterior vertebral height-based morphological grading system. This tool allowed the intuitive classification of fracture severity and demonstrated parallel improvements in both groups. The proportion of high-grade fractures decreased postoperatively, and no statistically significant difference was observed in the final grade distribution (*p* = 0.15; Table 6). These results support the sufficiency of biologically driven remodeling under stable fixation, regardless of fusion, and reinforce the applicability of this grading framework in future comparative studies [41,49]. Although several biomechanical/clinical studies associate >40% anterior height loss with anterior column insufficiency, this threshold is not part of the TLICS scoring system; in our study, TLICS (≥4) guided operative indication, whereas the anterior height-based grading was used as a supplementary severity descriptor.

Morphological restoration—particularly of anterior and posterior vertebral body height—has been widely recognized as a clinically relevant endpoint in the management of burst fractures [50,51]. Numerous studies have shown that restoring vertebral shape and height not only improves mechanical load distribution but also correlates with better functional outcomes and lower rates of progressive kyphosis or adjacent segment degeneration [42]. In our study, the majority of patients in both the PLF and PPF groups showed either improvement or maintenance of morphology grade postoperatively (PLF: 31/33; PPF: 27/28), as depicted in Figure 5. In this context, our finding that the morphological grade improved significantly in both groups, with no significant intergroup difference, has notable clinical implications. This suggests that even without formal fusion or extensive construct manipulation, PPF can support meaningful vertebral remodeling to a degree comparable to that of open techniques.

Radiographic outcomes may also be partially influenced by the fixation strategy. PLF more frequently utilized extended multilevel constructs, including two or more vertebrae above (63.6% vs. 28.6%, *p* = 0.03) and below (54.5% vs. 17.9%, *p* = 0.04) the fractures (Table 2). A wider fixation base provides increased leverage for angular correction, especially when combined with rod-based mechanical forces [52]. In contrast, PPF employs shorter, fracture-centered constructs, with alignment determined by segmental positioning and intraoperative biomechanics [34]. Despite these procedural differences, PPF achieved radiologic outcomes comparable to those of PLF, supporting its adequacy even without extended constructs or formal fusion. This highlights the potential of percutaneous fixation in providing sufficient sagittal control through minimal instrumentation, reinforcing its role as a standalone treatment option for thoracolumbar burst fractures.

Taken together, the observed radiological equivalence may reflect not only the surgical technique but also the intrinsic biological potential of the spine for remodeling and stabilization. Spontaneous facet joint arthrodesis begins with subchondral bone exposure and develops into a ring-like fusion mass within 6–12 months [53,54]. Concurrently, anterior column healing progresses from woven bone deposition, with structural stiffness typically restored by four weeks [55] to trabecular remodeling over 12–18 months. These dynamics align with our clinical protocol, which maintains the implants for at least 12 months. Biomechanical literature suggests that load sharing begins at eight weeks postoperatively, reducing hardware stress [55]. These findings suggest that formal PLF may not always be necessary when biological healing is adequately supported by instrumentation [53,56].

This observation aligns with prior studies comparing open fusion with standalone screw fixation, which have suggested that formal fusion does not significantly enhance outcomes when stable instrumentation is already achieved [57]. Given that PPF is fundamentally a screw-based fixation technique—albeit via a percutaneous route—the absence of a significant difference in radiological and morphological outcomes between PLF and PPF in our study is consistent with these earlier findings [58]. Indeed, this supports the broader notion that the benefits traditionally attributed to fusion may, in many cases, be attributable primarily to the mechanical effects of pedicle screw fixation itself [55,57]. This conceptual continuity was also emphasized in prior clinical discussions and presentations, further underscoring the interpretive framework of our results.

Complication rates were comparable between the groups. Screw loosening (24.2% vs. 17.9%, *p* = 0.54), revision surgery (3.0% vs. 7.1%, *p* = 0.46), and implant removal events did not differ significantly between the groups (Table 3). All removals were elective and unrelated to mechanical failure, indicating the long-term stability of both constructs. Notably, the absence of wound infections in the PPF group further supports the protective effect of muscle-preserving techniques, which may preserve the paraspinal innervation and contribute to faster recovery.

Collectively, these findings underscore the clinical viability of PPF as an effective alternative to open fusion for the treatment of thoracolumbar burst fractures. By achieving comparable alignment and morphological restoration with fewer surgical burdens, PPF may be a favorable strategy. Our anterior height-based morphological grading system demonstrated parallel improvements in both groups, offering a descriptive framework to track surgical impact on vertebral remodeling. However, this system is exploratory and requires further validation in larger, multicenter studies before its broader applicability can be confirmed.

This study has several limitations. First, it was conducted as a retrospective, single-center analysis with a relatively small sample size. Such a design inherently carries the risk of selection and information bias, and the limited cohort size reduces statistical power, particularly for subgroup analyses. These factors inevitably constrain the external validity and generalizability of our findings to other institutions and broader patient populations. In addition, the choice between posterolateral fusion (PLF) and percutaneous posterior fixation (PPF) was not determined by a standardized severity-based algorithm but was instead influenced by evolving practice patterns during the study period. Earlier in the study period, PLF was more frequently employed as the conventional open technique, whereas PPF became increasingly adopted in later years as minimally invasive methods gained broader acceptance. This temporal factor may have introduced selection bias, further limiting the external validity of our conclusions. Second, validated patient-reported outcome measures (PROMs), such as VAS and ODI, were not collected in this retrospective trauma cohort. As a result, we were unable to establish a direct clinico-radiographic correlation or comprehensively assess patient-centered recovery. Although previous studies have shown that restoration of vertebral height and sagittal alignment often parallels symptomatic improvement, such radiographic surrogates cannot substitute for PROMs. Moreover, our analysis did not include other clinical endpoints such as pain control or functional recovery, which could have provided further context regarding the potential advantages of minimally invasive approaches. Future multicenter studies incorporating standardized PROMs will therefore be essential to strengthen the clinical relevance of radiological outcomes. Third, although we defined the 24-month postoperative visit as the final follow-up, this duration represents early to mid-term rather than true long-term results. While both groups demonstrated comparable improvements in sagittal alignment and vertebral morphology over this period, we did not specifically investigate late complications such as progressive kyphosis, adjacent segment degeneration (ASD), or proximal junctional kyphosis (PJK). These issues are particularly relevant in non-fusion constructs such as PPF, where long-term durability remains a central concern. We acknowledge that important differences between PPF and PLF could emerge over extended follow-up, and additional longitudinal studies will be required once sufficient data become available. Fourth, although all procedures were performed by a single experienced surgeon, subtle intraoperative variations and surgeon-specific decision-making may have introduced bias. While this consistency minimizes variability in technique, it simultaneously limits the generalizability of our results across different surgical practices. Finally, although our anterior height-based morphological grading system demonstrated promising applicability for classifying fracture severity and evaluating postoperative restoration, it requires further validation in larger, multicenter, prospective studies. Broader adoption of such a grading framework could facilitate more standardized comparisons across future trials and contribute to the development of more consistent treatment guidelines.

## 5. Conclusions

This study demonstrated that percutaneous posterior screw fixation without formal fusion provided radiological and morphological outcomes comparable to those of traditional open posterolateral fusion for thoracolumbar burst fractures. Despite the absence of grafting, percutaneous fixation effectively preserved global sagittal alignment and achieved similar anterior vertebral height restoration. Our anterior height-based morphological grading system further confirmed equivalent postoperative recovery between groups. These findings suggest that percutaneous fixation alone, which minimizes the surgical burden while ensuring structural stability, represents a clinically effective and biologically sound alternative to open fusion in selected patients.

## Figures and Tables

**Table 1:** Demographic and clinical characteristics of patients.

	PLF (*n* = 33)	PPF (*n* = 28)	*p*
**Demographic characteristics**			
Age (years)	66.64 ± 11.99	65.71 ± 16.98	0.81
Sex (M:F)	11:22	11:17	0.63
BMI (kg/m^2^)	22.15 ± 3.82	23.53 ± 3.96	0.17
**Clinical Characteristics**			
BMD (T-score)	−1.48 ± 1.46	−0.37 ± 3.11	0.12
CKD, *n* (%)	4 (12.1%)	0 (0.0%)	0.06
Osteoporosis medication before Fracture, *n* (%)			0.53
None	30 (90.9%)	25 (89.3%)	
Prolia	3 (9.1%)	2 (7.1%)	
Forsteo	0 (0.0%)	1 (3.6%)	
Osteoporosis medication after Fracture, *n* (%)			0.23
None	20 (60.6%)	20 (71.4%)	
Prolia	10 (30.3%)	5 (17.9%)	
Forsteo	2 (6.1%)	3 (10.7%)	
Zoledronic Acid	4 (12.1%)	0 (0.0%)	

**Abbreviations:** BMD, bone mineral density; BMI, body mass index; CKD, chronic kidney disease; Prolia, denosumab; Forsteo, teriparatide; Zoledronic acid, intravenous bisphosphonate.

**Table 2:** Comparison of fracture level, fixation, and posterior column disruption.

	PLF (*n* = 33)	PPF (*n* = 28)	*p*
**Fracture level**			0.99
T11	4 (12.1%)	3 (10.7%)	
T12	9 (27.3%)	8 (28.6%)	
L1	14 (42.4%)	12 (42.9%)	
L2	6 (18.2%)	5 (17.9%)	
**Upper fixation, *n* (%)**			0.03 *
3	2 (6.1%)	1 (3.6%)	
2	19 (57.6%)	7 (25.0%)	
1	11 (33.3%)	20 (71.4%)	
0	1 (3.0%)	0 (0.0%)	
**Lower fixation, *n* (%)**			0.04 *
4	1 (3.0%)	0 (0.0%)	
3	2 (6.0%)	1 (3.6%)	
2	15 (45.5%)	4 (14.3%)	
1	15 (45.5%)	21 (75.0%)	
0	0 (0.0%)	2 (7.1%)	
**Fracture level fixation, *n* (%)**	24 (72.7%)	28 (100.0%)	<0.01 *
**Posterior Column Disruption, *n* (%)**	10 (30.3%)	13 (46.4%)	0.19
PLC injury, *n* (%)	8 (24.2%)	6 (21.4%)	
Spinous process fracture, *n* (%)	3 (9.1%)	9 (32.1%)	

* *p*-value < 0.05. Notes: The fracture-level fixation indicated whether the fractured vertebrae were included in the fixation construct. Upper and lower fixations refer to the number of vertebral levels fixed above and below the fractured level, respectively.

**Table 3:** Perioperative outcomes and complications between the two groups.

	PLF (*n* = 33)	PPF (*n* = 28)	*p*
**Perioperative parameters**			
OP time (minute)	205.79 ± 66.31	160.71 ± 52.65	<0.01 *
Estimated blood loss (cc)	317.27 ± 189.48	165.00 ± 13.74	<0.01 *
Hospital day (days)	16.27 ±13.03	11.00 ± 6.99	0.05 *
**Complications**			
Screw loosening, *n* (%)	8 (24.2%)	5 (17.9%)	0.54
Revision, *n* (%)	1 (3.0%)	2 (7.1%)	0.46
Prominence, *n* (%)	3 (9.1%)	0 (0.0%)	0.10
**Implant removal, *n* (%)**	6 (18.2%)	5 (17.9%)	0.97

* *p*-value < 0.05.

**Table 4:** Comparison of radiological outcomes between the two groups.

		PLF (*n* = 33)	PPF (*n* = 28)	*p*
LKA (°)				
	Pre	15.25 ± 15.01	11.57 ± 17.51	0.39
	Post	9.52 ± 5.70	9.21 ± 15.32	0.92
	Post-Pre	−7.77 ± 9.96	−1.53 ± 7.60	0.01 *
WA (°)				
	Pre	12.59 ± 8.51	14.27 ± 8.41	0.45
	Post	5.14 ± 7.82	7.42 ± 4.77	0.20
	Post-Pre	−7.72 ± 7.93	−6.79 ± 6.70	0.64
PI (°)				
	Pre	47.98 ± 10.81	52.62 ± 9.68	0.12
	Post	52.12 ± 11.50	52.51 ± 9.45	0.90
	Post-Pre	4.25 ± 14.87	1.45 ± 10.18	0.49
LL (°)				
	Pre	34.73 ± 22.43	38.52 ± 12.04	0.43
	Post	35.22 ± 22.49	36.02 ± 12.12	0.44
	Post-Pre	−0.11 ± 29.27	−2.25 ± 10.04	0.73
PI−LL (°)				
	Pre	11.08 ± 23.17	10.70 ± 14.43	0.72
	Post	14.94 ± 24.69	11.36 ± 11.81	0.38
	Post-Pre	3.87 ± 30.12	0.66 ± 18.28	0.15
SVA (mm)				
	Pre	86.35 ± 9.45	87.44 ± 9.79	0.66
	Post	43.91 ± 9.14	45.50 ± 9.76	0.51
	Post-Pre	−42.44 ± 12.84	−41.95 ± 14.38	0.89

* *p*-value < 0.05. Notes: Lumbar lordosis is represented as lordotic angles in the table. Abbreviations: LKA, local kyphotic angle; WA, wedge angle; PI, pelvic incidence; LL, lumbar lordosis; SVA, sagittal vertical axis.

**Table 5:** Comparison of anterior and posterior vertebral body height ratios on radiographs between the two groups.

		PLF (*n* = 33)	PPF (*n* = 28)	*p*
Anterior body height (%)				
	Pre	60.85 ± 24.89	66.49 ± 14.66	0.28
	Post	76.57 ± 27.36	78.99 ± 11.38	0.65
	Post-Pre	16.24 ± 29.52	12.90 ± 14.29	0.58
Posterior body height (%)				
	Pre	83.07 ± 17.00	89.45 ± 26.75	0.27
	Post	79.70 ± 21.20	90.24 ± 9.00	0.02 *
	Post-Pre	−3.52 ± 24.76	0.94 ± 24.46	0.50

* *p*-value < 0.05.

**Table 6:** Comparison of preoperative and postoperative vertebral fracture grades between the two groups.

		PLF (*n* = 33)	PPF (*n* = 28)	*p*
**Pre-OP Fracture Grade, *n* (%)**				0.17
	I	5 (15.2%)	3 (10.7%)	
	II	11 (33.3%)	16 (57.1%)	
	III	17 (51.5%)	9 (32.1%)	
**Post-OP Fracture Grade, *n* (%)**				0.15
	I	15 (45.5%)	13 (46.4%)	
	II	10 (30.3%)	13 (46.4%)	
	III	8 (24.2%)	2 (7.1%)	

## Data Availability

The datasets used and/or analyzed in the current study are available from the corresponding author upon reasonable request.

## Abbreviations

The following abbreviations are used in this manuscript:

PPF	Percutaneous Posterior Fixation
PLF	Posterolateral Fusion
LKA	Local Kyphotic Angle
TLICS	Thoracolumbar Injury Classification and Severity Score
